# Abalone farm discharges the withering syndrome pathogen into the wild

**DOI:** 10.3389/fmicb.2013.00373

**Published:** 2013-12-06

**Authors:** Kevin D. Lafferty, Tal Ben-Horin

**Affiliations:** ^1^Western Ecological Research Center – US Geological Survey, UCSB Marine Science Institute, Santa BarbaraCA, USA; ^2^Bren School of Environmental Science and Management, University of CaliforniaSanta Barbara, CA, USA; ^3^Institute of Marine and Coastal Sciences, Rutgers, The State University of New JerseyNew Brunswick, NJ, USA

**Keywords:** abalone, aquaculture, emerging disease, endangered species, fisheries, pathogen, water quality

## Abstract

An intracellular bacterium Candidatus *Xenohaliotis californiensis*, also called Withering-Syndrome Rickettsia-Like Organism (WS-RLO), is the cause of mass mortalities that are the chief reason for endangerment of black abalone (*Haliotis cracherodii*). Using a real-time PCR assay, we found that a shore-based abalone farm (AF) in Santa Barbara, CA, USA discharged WS-RLO DNA into the ocean. Several other shore-based AFs discharge effluent into critical habitat for black abalone in California and this might affect the recovery of wild black abalone. Existing regulatory frameworks exist that could help protect wild species from pathogens released from shore-based aquaculture.

## INTRODUCTION

Black abalone (*Haliotis cracherodii*) were once stacked three deep in the California intertidal (Cox, 1960). But in the mid-1980s, marine biologists noticed black abalone disappearing from their study plots (Haaker et al., 1992). Lying next to the empty quadrats were dying abalone, their muscular foot withered and unable to remain attached to the rocks. These “withering syndrome” die offs were often rapid and extensive (Richards and Davis, 1993). At first, the die offs were attributed to an El Niño Southern Oscillation event (Davis et al., 1992), but the continued spread of mass mortalities was more indicative of an infectious process (Lafferty and Kuris, 1993). Pathologists discovered the cause: a novel intracellular bacterium Candidatus *Xenohaliotis californiensis*, or Withering Syndrome Rickettsia-Like Organism (WS-RLO; Friedman et al., 2000). Withering syndrome spread north and south from the Santa Barbara Channel Islands over the next two decades throughout most of the black abalone’s range (Lafferty and Kuris, 1993; Altstatt et al., 1996). WS-RLO also infects and impacts, to various degrees, the six other California abalone species (Friedman et al., 2002). The pathogen can spill over from more resistant abalone species, which could contribute to the decline of less resistant species such as black abalone (Ben-Horin, 2013). Locally extirpated from most of its range with no sign of population recovery, the National Marine Fisheries Service listed the black abalone as endangered in 2009 (Neuman et al., 2010). With no commercial fishery, prices for red abalone (*Haliotis rufescens*) increased and shore-based abalone farms (AF) grew and sold four to 5-year-old juvenile red abalone. By 2008, production was dominated by 3–4 farms (Moore and Moore, 2008) and had risen to ~227 tonnes (US $8–9 million), a production level that remains true today.

Even though the young abalone were soon infected with WS-RLO in culture, the prolonged incubation period and temperature dependence of clinical withering syndrome (Moore et al., 2011) allows farms to keep infected abalone alive to market size with minimal losses. Farms in cooler regions, such as central California, suffer lower losses despite the presence of the pathogen (Friedman and Finley, 2003). Pathogen export from AFs has two precedents that motivated our study. In the early 1990s, California AFs became infested with a sabellid polychaete worm (*Terebrasabella heterouncinata,* Fitzhugh and Rouse, 1999) that abalone farmers introduced along with abalone from South Africa. The worms live in snail shells and their presence slows growth (Kuris and Culver, 1999). Shell debris exiting the outflow of The Abalone Farm at Cayucos, California, led to the infestation of native turban snails (Kuris and Culver, 1999). However, counter to most species introductions, the sabellid-worm invasion was halted (Culver and Kuris, 2000). When the discharge was discovered, volunteers removed 1.6 million snails from around the farm, ceasing transmission in the wild. The AF then cleaned up its stock, screened its outflow, and stopped dumping shell debris into the intertidal. The second precedent is from Australia where, in 2005, a herpes virus that causes Abalone Viral Ganglioneuritis led to mortalities in AFs in Victoria. In 2006, the disease emerged in wild abalone near farms where it has spread along the coast, leading to substantial fisheries losses (Hooper et al., 2007); nowhere is the conflict between aquaculture and wild fisheries more clear (and litigious). Global declines in wild fisheries and the rise in demand for seafood for human consumption have driven the rapid growth of aquaculture, particularly for expensive foods like abalone (Naylor et al., 2000). Growth in aquaculture may reprieve pressure on wild fisheries; however, ecological risks such as the spread of infectious diseases from farms to wild stocks have uncertain and often negative impacts on wild populations (Daszak et al., 2001). Understanding the indirect impacts of aquaculture is therefore critical for the sustainable management of disease-impacted fisheries. Here we asked whether the WS-RLO pathogen was being exported from an abalone aquaculture facility near Santa Barbara, CA, USA. A real-time, quantitative polymerase chain reaction (qPCR) test for the pathogen in water samples indicated high WS-RLO DNA densities at the farm’s outflow that dissipated with distance.

## MATERIALS AND METHODS

Seawater samples were collected for assessment of WS-RLO in the western Santa Barbara Channel (CA, USA) during August 2011. We first collected three 100 ml samples directly from the effluent of the AF. We then collected 36 100 ml seawater samples immediately offshore the AFs at Dos Pueblos (DP) and again at Hope Ranch-More Mesa (HR-MM), located 20 km ESE of the farm. Both near shore sites contain patchy rocky subtidal habitat within soft bottom substrate. At both sites, 18 of the 36 samples were collected at the surface and the remaining samples were collected at a depth of approximately 5 m. We collected 9 of the 18 surface and subtidal samples at both sites over rocky habitat and the remaining nine samples were collected over soft bottom habitat. After collection, the seawater samples were placed on ice in a cooler and kept in the dark until further processing in the laboratory. Seawater samples were filtered onto 47 mm diameter, 0.2 μm-pore size, Supore^®^ filters (Pall Corp., Port Washington, NY, USA). We rinsed the filter apparatus with 10% bleach between each sample to prevent carry-over of WS-RLO, and filtered four, 100 ml artificial seawater samples as negative controls (negative filter controls). Filters were folded and placed immediately into microcentrifuge tubes containing 200 μl of Qiagen lysis buffer with proteinase K (Qiagen, Santa Clarita, CA, USA). We isolated DNA from the filters using a Qiagen DNeasy Stool Kit following the manufacturer’s protocol, which included a final elution of the DNA from the column using 100 μl of the elution buffer AE (Qiagen). We quantified the density of WS-RLO DNA in seawater samples by real-time SYBR Green^®^ PCR on the Applied Biosystems 7500 Fast Real-Time PCR system (Life Technologies, Carlsbad, CA, USA), using the primer pair RA5-1 and RA3-6 specific to a 160 bp fragment of the WS-RLO 16S rDNA (GenBank Accession number: AF133090) described by Andree et al. (2000). The WS-RLO specific primer mix was prepared manually by diluting each 100 μM primer (Life Technologies) to 9 μM. Amplifications were done in 20 μL reaction mixtures with 20–50 ng of genomic DNA measured spectrophotometrically. Reagents were added in the following proportions: 10 μL Fast SYBR Green Master Mix with AmpliTaq^®^ Fast DNA Polymerase (Life Technologies), 2 μL RA5-1, 2 μL RA3-6, 4 μL nuclease-free H_2_0 (Life Technologies), and 2 μL of template DNA for a 20 μL reaction volume. The thermal-cycling conditions were as follows: initial polymerase activation for 20 s at 95°C, followed by 50 cycles of 3 s at 95°C, and 30 s at 60°C.

Quantification of the amplified product was measured on a cycle-by-cycle basis via the acquisition of a fluorescent signal generated by the binding of the SYBR Green^®^ fluorophore to double stranded DNA. Our interest was in the relative differences in concentrations of WS-RLO DNA among sampling locations, and therefore used a relative standard curve for DNA quantification as follows. First, WS-RLO DNA was isolated from post-esophagus tissue of three WS-RLO positive red abalone using a Qiagen DNeasy tissue kit following the manufacturer’s protocol. The obtained DNAs were pooled to provide a 1× relative density sample, then 10-fold serially diluted using the elution buffer AE (Qiagen). We prepared three assays of 20 replicates of each relative density standard (*D*_S_) and these relative standard samples were analyzed by real-time PCR. The 7500 Fast System software fits a standard curve by regression of *D*_S_ on the threshold cycle number, *C*_t_, at which the fluorescence signal (Δ*R*_n_) crosses a value exceeding the background fluorescence. This *C*_t_ value is proportional to the logarithm of the target DNA concentration in the assay. We set the threshold value of Δ*R*_n_ to 0.1, based on previous runs of the 7500 Fast System. We determined mean values of *C*_t_, as well as the associated standard deviations, for all values of *D*_S_. The sensitivity of real-time PCR assays to differences in initial template amounts increases at low concentrations of template DNA, and we therefore defined the limit of detection (LoD) as the minimum value of *D*_S_ where ≥50% of the test runs amplified and the standard deviation of estimated *C*_t_ values ≤ 0.5 (OIE-World Organisation for Animal Health, 2009). Unknown seawater samples were run in triplicate, and all runs included triplicate relative standards, two negative filter controls and two negative plate controls (nuclease-free H_2_O in lieu of DNA template). The 7500 Fast System software automatically plots the relationship between relative density and *C*_t_ and converts *C*_t_ values of unknown samples to relative densities using the obtained regression formula. We confirmed the specificity of all amplified products by melting curve analysis. Precision, or reproducibility of the real-time SYBR Green^®^ assay, was estimated in a separate experiment. Reproducibility can vary within an assay (intra-assay variability), due to reaction-to-reaction variance in pipetting volumes and measurement among wells, as well as between assays (inter-assay variability) due to variance arising from slight differences in reaction components. We tested the precision of the real-time assay in three separate runs, sampling five replicates of one WS-RLO positive seawater sample and the 1× relative standard sample in each run. We sampled intra-assay variability as the standard deviation of the estimated *C*_t_ values within each run, and tested the equality of variances between runs using Levene’s tests. We tested for inter-assay variability using ANOVA. We estimated the relative concentration index (*CI*_R_) for all seawater samples as the mean of obtained values of relative density among triplicate samples, and tested for differences in *CI*_R_ among the habitat and depth covariates, as well as among the sampling sites by Kruskal–Wallis analysis of variance by ranks. All statistical analyses were performed in Matlab^®^ version 8.1.0 (The Mathworks, Inc., Natick, MA, USA).

## RESULTS

**FIGURE 1:**
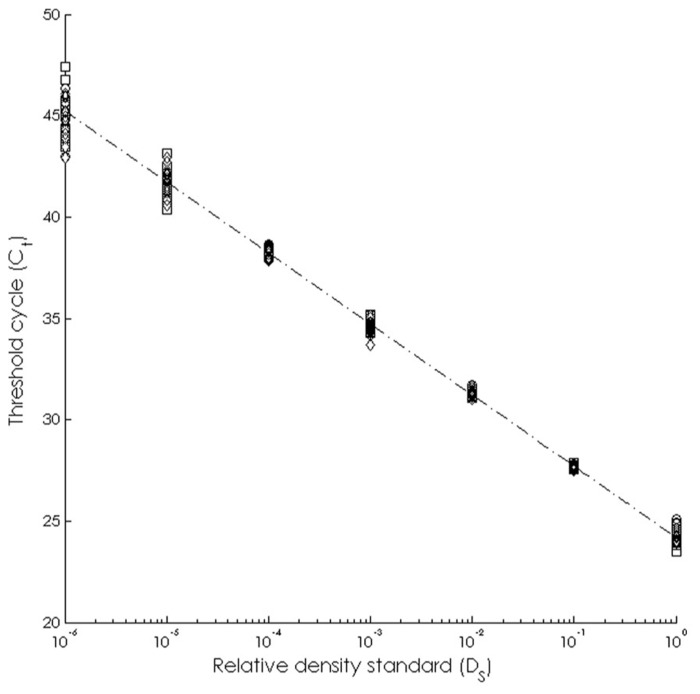
**Standard curve representing the relationship between the relative concentration of the WS-RLO DNA (*D*_**S**_) and the threshold cycle (*C*_**t**_) determined by the real-time qPCR assay**.

**FIGURE 2:**
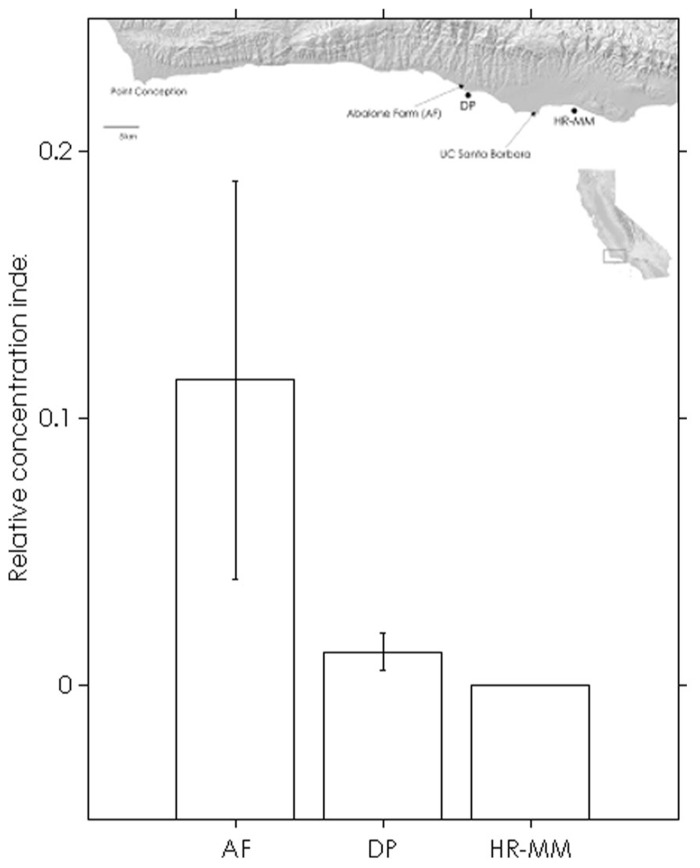
**Means (±SE) of WS-RLO DNA relative concentration indices at the abalone farm (AF), Dos Pueblos (DP), and Hope Ranch-More Mesa (HP-MM), with a map of the sites.** Long-shore current flows from left to right. The index describes the concentration of WS-RLO DNA in seawater relative to pooled sample of WS-RLO DNA taken from the post-esophagus of WS-RLO infected red abalone with >100 WS-RLO inclusions per 200× magnification field of view.

The assay was able to detect dilute WS-RLO DNA in relative standard and seawater samples, and both filter and plate negative controls tested negative for WS-RLO DNA. We observed amplification in ≥50% of the standard samples across seven orders of magnitude in the three replicate assays (**Figure 1**), and all but three points fell within the 95% confidence interval of *C*_t_ estimated for each relative standard. The standard deviation of *C*_t_ values in the 0.00001× and 0.000001× standards exceeded the *a priori* threshold value of 0.5 however, and we therefore used a detection limit of 0.0001× (mean *C*_t_ = 38.27 cycles). The efficiency of the assay, estimated from the slopes of the three replicate standard curve regression equations ranged from 92 to 93%. The assay was repeatable enough to give us confidence that variation among samples was attributed to variation in the signal. For all intra-assay variability tests of real-time PCR assay precision, the standard deviation of the estimated *C*_t_ values within each run was ≤0.15. We did not find significant differences in the variability of estimated *C*_t_ values between runs, for both the 1× relative standard sample (Levene’s test; *P* = 0.34) and positive seawater sample (Levene’s test; *P* = 0.38). Estimated *C*_t_ values did not differ between runs, both for the 1× relative standard sample (one-way ANOVA; *F*_2,14_ = 1.41, *P* = 0.28) and the positive seawater sample (one-way ANOVA; *F*_2,14_ = 0.66, *P* = 0.54). WS-RLO DNA dissipated with distance from the farm. All seawater samples taken at the farm effluent contained WS-RLO DNA. Seventeen of 36 samples taken immediately offshore the farm at DP contained WS-RLO DNA, and only one of 36 samples taken at Hope Ranch/More Mesa contained WS-RLO DNA. We did not observe significant associations between relative concentration indices and habitat (Kruskal–Wallis test; *H* = 0.54, df = 1, *P* = 0.63) and depth (Kruskal–Wallis test; H = 3.48, df = 1, *P* = 2.21). However, relative concentration indices differed significantly among the sampling locations (Kruskal–Wallis test; H = 77.18, df = 2, *P* < 0.001), and were greatest at the farm outflow and dissipated in the seawater offshore from the aquaculture facility (**Figure 2**). WS-RLO DNA was detectable, but very dilute at Hope Ranch/More Mesa.

## DISCUSSION

Our results show that outflow from a California abalone aquaculture facility contains WS-RLO DNA. This DNA also occurs offshore of the outflow and is distributed throughout the water column over sand and rock habitat. The DNA is dilute, but detectable for almost 20 km to the east (with the long-shore current). There are other potential sources of the dilute WS-RLO DNA detected in our eastern sampling site. Wild abalone could exist there undetected and several dozen infected abalone are housed in the flow-through sea water system at the University of California, Santa Barbara (UCSB), CA, USA, though outflow from these abalone is not discharged directly into the ocean (see below). How WS-RLO discharge affects black abalone recovery depends on whether AFs are near good black abalone habitat. Black abalone are most common at exposed rocky shores with vertical rock faces and crevices (Cox, 1960). This habitat is abundant in northern Santa Barbara County, and on the offshore Channel Islands. However, due to low relief rocky habitat and frequent burial by sand, long-time UCSB collector Shane Anderson (personal communication) only saw black abalone regularly at two places in our study area: in the mid 1970s to mid 1990s there were three, >10 cm black abalone on the most offshore rock at Campus Point (near the campus seawater intake), and black abalone were common nestled amongst the mussels on the Ellwood Pier (4 km east of the Cultured AF) until the mid 1990s. Overall, these observations suggest that the area near the AF we sampled is not particularly good black abalone habitat. In fact, southern Santa Barbara County was not designated as black abalone critical habitat. Although the AF we sampled is not located in critical habitat for black abalone, there are AFs in black abalone critical habitat. If these farms also discharge WS-RLO, they might prevent black abalone from recolonizing into critical habitat. The only way to understand the magnitude of pathogen discharge is to test the outflow of other facilities that house abalone. A complication in determining the impact of pathogen discharge is the recent discovery of a phage that infects the WS-RLO (Friedman and Crosson, 2012). This phage appears to be common in AFs and could explain reductions in the impact of WS-RLO on farmed abalone. The export of phage from AFs might also help reduce the impact of WS-RLO on wild abalone, though this remains a hypothesis. The impact of the WS-RLO from AFs also depends on how far currents can transport live, viable WS-RLO from the discharge. The WS-RLO can likely survive in seawater for several days based on the spread of the bacterium and osmolarity of host cells that is similar to that of seawater (C. Friedman pers. comm.). Ocean currents in the western Santa Barbara Channel have a general counterclockwise circulation, and therefore effluent from the sampled AF, as well as any presumptive pathogens contained within the effluent, flows toward critical habitat at San Miguel Island with an expected 80 km transit in about 5 days (Beckenback, 2004). Using the qPCR assay described, it should be possible to predict the advection and dilution of WS-RLO with distance from this and other AFs, and therefore quantify the indirect impact of aquaculture on wild abalone stocks. However, given that wild infected abalone may be present and that the qPCR assay tests for presence of target DNA and not the presence of a viable organism (Burreson, 2008), these data should be used to inform transmission trials needed to confirm this hypothesis. The qPCR assay, however, serves as a proxy to assess potential risk. Regulators and environmental advocates have not considered the potential impact of WS-RLO discharge from AFs, research institutions, or public aquaria. For instance, the Monterey Bay Aquarium’s Seafood Watch program labeled California farmed abalone as sustainable seafood because the main known environmental effects were assumed to be minor now that farms no longer export sabellid worms. For AFs outside of black abalone critical habitat, farm discharge may not present an impact requiring management (depending on how far the WS-RLO can travel). For AFs that discharge into critical habitat, one possible management option is to eliminate the pathogen from the system by using antibiotics to treat stock and prevent re-infection (Friedman et al., 2003). Although this method is labor intensive, treatment with the antibiotic oxytetracycline has shown to provide long-term protection from WS-RLO infection (Friedman et al., 2007), and therefore provides a feasible management option for small to medium-sized farms, public aquaria, and research institutions. An alternative to antibiotic treatment would be to divert the discharge from the ocean. For instance, outflow could be sent to a sewer or to holding ponds (e.g., artificial wetlands) instead of to the ocean. As an example, relative concentrations of WS-RLO DNA at our abalone facility at UCSB are comparable to the relative concentrations of WS-RLO DNA we observed at Hope Ranch – More Mesa (observed *CI*_R_ have ranged from 0 to 0.0002). To diminish the chance that WS-RLO will enter the open ocean, the outflow from our abalone facility is directed into the Campus Lagoon, an artificial estuary-like pond closed to tidal flushing. Another approach would be to treat the discharge with heating, chemicals, filtration, or UV sterilization. Further research could help assess the cost and benefits of each approach. An obvious question is if and how to regulate the discharge of WS-RLO. Whether or not to regulate is a policy decision that we will not advocate for here. In California, there are many agencies involved in fisheries and coastal development. The California Department of Fish and Wildlife (CDFW) can prohibit aquaculture discharge or place restrictions or requirements on aquaculture operations where it is determined aquaculture activities are detrimental to adjacent native wildlife. The California National Pollutant Discharge Elimination System (NPDES) considers that dischargers of point source pollutants shall not cause degradation of indigenous biota, and marine communities. The National Marine Fisheries Service (NMFS) could choose to determine that aquaculture discharge of WS-RLO jeopardizes the continued existence of endangered black abalone. Finally, construction of AFs requires a coastal development permit by the California Coastal Commission and this state agency has the flexibility to consider impacts to marine biological resources when granting permits. Decisions to enact this regulatory framework require information about potential impacts, such as we have described above. Our results focus on black abalone, but these general issues apply to aquaculture projects around the world, including for finfish. For example, wild juvenile salmon can be impacted by sea lice as they swim by salmon farms (Krkosek et al., 2006). Similarly salmon farms may have increased bacterial kidney disease (Jude and Leach, 1999). Regulators might specifically consider whether discharge from aquaculture facilities includes pathogens that can affect wild species, particularly those that are in danger of extinction.

## Conflict of Interest Statement

The authors declare that the research was conducted in the absence of any commercial or financial relationships that could be construed as a potential conflict of interest.

## References

[B1] Altstatt J. M. Ambrose R. F. Engle J. M. Haaker P. L. Lafferty K. D. Raimondi P. T. (1996). Recent declines of black abalone *Haliotis cracherodii* on the mainland coast of central California. *Mar. Ecol. Prog. Ser.* 142 185–192 10.3354/meps142185

[B2] Andree K. B. Friedman C. S. Moore J. D. Hedrick R. P. (2000). A polymerase chain reaction assay for the detection of genomic DNA of a rickettsiales-like prokaryote associated with withering syndrome in black abalone (*Haliotis cracherodii*). *J. Shellfish Res.* 19 213–218

[B3] Beckenback E. H. (2004). *Surface Circulation in the Santa Barbara Channel: an Application of High Frequency Radar for Descriptive Physical Oceanography in the Coastal Zone*. Santa Barbara: University of California

[B4] Ben-Horin T. (2013). *Withering Syndrome and the Management of Southern California Abalone Fisheries*. Santa Barbara: University of California

[B5] Burreson E. M. (2008). Misuse of OCR assay for the diagnosis of mollusc protistan infections. *Dis. Aquat. Organ* 80 81–83 10.3354/dao01925 18714688

[B6] Cox K. W. (1960). Review of the abalone of California. *Calif. Fish and Game* 46 381–406

[B7] Culver C. S. Kuris A. M. (2000). The apparent eradication of a locally established introduced marine pest. *Biological Invas.* 2 245–253 10.1023/A:1010082407254

[B8] Daszak P. Cunningham A. A. Hyatt A. D. (2001). Anthropogenic environmental change and the emergence of infectious diseases in wildlife. *Acta Trop.* 78 103–116 10.1016/S0001-706X(00)00179-0 11230820

[B9] Davis G. E. Richards D. V. Haaker P. L. Parker D. O. (1992). “Abalone population declines and fishery management in southern California,” in *Abalone of the World: Biology, Fisheries and Culture* eds Shepherd S. A. Tegner M. J. Gusman del Proo S. A. (Cambridge: Cambridge University Press) 237–249

[B10] Fitzhugh K. Rouse G. W. (1999). A remarkable new genus and species of fan worm (Polychaeta: Sabellidae: Sabellinae) associated with marine gastropods. *Invert. Biol.* 118 357–390 10.2307/3227007

[B11] Friedman C. S. Andree K. B. Beauchamp K. A. Moore J. D. Robbins T. T. Shields J. D. (2000). “Candidatus Xenohaliotis californiensis” a newly described pathogen of abalone, *Haliotis spp*., along the west coast of North America. *Int. J. Syst. Evol. Microbiol.* 50 847–855 10.1099/00207713-50-2-847 10758896

[B12] Friedman C. S. Biggs W. Shields J. D. Hedrick R. P. (2002). Transmission of withering syndrome in black abalone, *Haliotis cracherodii* Leach. *J. Shellfish Res.* 21 817–824

[B13] Friedman C. S. Crosson L. M. (2012). Putative phage hyperparasite in the Rickettsial pathogen of abalone, “*Candidatus Xenohaliotis* californiensis”. *Invertebr. Microbiol.* 64 1064–1072 10.1007/s00248-012-0080-4 22729142

[B14] Friedman C. S. Finley C. A. (2003). Anthropogenic introduction of the etiological agent of withering syndrome into northern California abalone populations via conservation efforts. *Can. J. Fish. Aquat. Sci.* 60 1424–1431 10.1139/f03-121

[B15] Friedman C. S. Trevelyan G. Robbins T. T. Mulder E. P. Fields R. (2003). Development of an oral administration of oxytetracycline to control losses due to withering in cultured red abalone *Haliotis rufescens*. *Aquaculture* 224 1–23 10.1016/S0044-8486(03)00165-0

[B16] Friedman C. S. Scott B. B. Estes-Strenge R. M. Vadopalas B. McCormick T. B. (2007). Oxytetracycline as a tool to manage and prevent losses of the endangered white abalone, *Haliotis sorenseni*, caused by withering syndrome. *J. Shellfish Res.* 26:877–885 10.2983/0730-8000(2007)26[877:OAATTM]2.0.CO;2

[B17] Haaker P. L. Richards D. V. Friedman C. S. Davis G. E. Parker D. O. Togstad H. A. (1992). “Mass mortality and withering syndrome in black abalone, *Haliotis cracherodii* in California,” in *Abalone of the World: Biology, Fisheries and Culture*. S. eds A. Shepherd, M. J. Tegner, S. A. Gusman del Proo (Cambridge: Cambridge University Press) 214–224

[B18] Hooper C. Hardy-Smith P. Handlinger J. (2007). Ganglioneuritis causing high mortalities in farmed Australian abalone (*Haliotis laevigata* and *Haliotis rubra*). *Aust. Vet. J.* 85 188–193 10.1111/j.1751-0813.2007.00155.x 17470067

[B19] Jude D. J. Leach J. (1999). “Great lakes fisheries,” in *Inland Fisheries Management in North America* eds Kohler C. A. Hubert W. A. (Quebec city: American Fisheries Society) 623–664

[B20] Krkosek M. Lewis M. A. Morton A. Frazer L. N. Volpe J. P. (2006). Epizootics of wild fish induced by farm fish. *Proc. Natl. Acad. Sci. U.S.A.* 103 15506–15510 10.1073/pnas.0603525103 17021017 PMC1591297

[B21] Kuris A. M. Culver C. S. (1999). An introduced sabellid polychaete pest infesting cultured abalones and its potential spread to other California gastropods. *Invertebr. Biol.* 118 391–403 10.2307/3227008

[B22] Lafferty K. D. Kuris A. M. (1993). Mass mortality of abalone *Haliotis cracherodii* on the California channel islands: tests of epidemiological hypotheses. *Mar. Ecol. Progr. Ser.* 96 239–248 10.3354/meps096239

[B23] Moore J. D. Marshman B. C Chun C. S. Y. (2011). Health and survival of red abalone *Haliotis rufescens* from San Miguel Island, California, USA, in a laboratory simulation of La Niña and El Niño conditions. *J. Aquat. Anim. Health* 23 78–84 10.1080/08997659.2011.568860 21834330

[B24] Moore T. O. Moore J. D. (2008). Culture of Abalone, *Haliotis* spp. *Calif. Depart. Fish Game Annu. Stat. Fish. Rep.* 18 1–6

[B25] Naylor R. L. Goldburg R. J. Primavera J. H. Kautsky N. Beveridge M. C. Clay J. (2000). Effect of aquaculture on world fish supplies. *Nature* 405 1017–1024 10.1038/35016500 10890435

[B26] Neuman M. Tissot B. Blaricom G. V. (2010). Overall status and threats assessment of black abalone (*Haliotis cracherodii* Leach, 1814) populations in California. *J. Shellfish Res.* 29 577–584 10.2983/035.029.0305

[B27] OIE-World Organisation for Animal Health. (2009). “Principles and methods of validation of diagnostic assays for infectious diseases,” in *Manual of Diagnostic Tests for Aquatic Animals*, 6th Edn, eds. B. Vallat and E. M. Bernoth (Paris: Office International Des Epizooties) 10–20

[B28] Richards D. V. Davis G. E. (1993). Early warnings of modern population collapse in black abalone Hallotis cracherodii, Leach 1814 at the California Channel Islands. *J. Shellfish Res.* 12 189–194

